# Incentives to change: effects of performance-based financing on health workers in Zambia

**DOI:** 10.1186/s12960-017-0179-2

**Published:** 2017-02-28

**Authors:** Gordon C. Shen, Ha Thi Hong Nguyen, Ashis Das, Nkenda Sachingongu, Collins Chansa, Jumana Qamruddin, Jed Friedman

**Affiliations:** 1 Department of Health Policy and Management, Graduate School of Public Health and Health Policy, City University of New York, 55 West 125 Street Room 806, New York, NY 10027 United States of America; 2 Health, Nutrition, and Population Global Practice, The World Bank Group, 1818 H Street, NW, Washington, DC 20433 United States of America; 3 Department of Gender Studies, University of Zambia, P.O. Box 32379, Lusaka, Zambia; 4 Development Research Group, The World Bank Group, Washington, DC United States of America

**Keywords:** Performance-based financing, Pay-for-performance, Organizational behavior, Mixed methods, Human resources for health, Health system strengthening, Zambia

## Abstract

**Background:**

Performance-based financing (PBF) has been implemented in a number of countries with the aim of transforming health systems and improving maternal and child health. This paper examines the effect of PBF on health workers’ job satisfaction, motivation, and attrition in Zambia. It uses a randomized intervention/control design to evaluate before–after changes for three groups: intervention (PBF) group, control 1 (C1; enhanced financing) group, and control 2 (C2; pure control) group.

**Methods:**

Mixed methods are employed. The quantitative portion comprises of a baseline and an endline survey. The survey and sampling scheme were designed to allow for a rigorous impact evaluation of PBF or C1 on several key performance indicators. The qualitative portion seeks to explain the pathways underlying the observed differences through interviews conducted at the beginning and at the three-year mark of the PBF program.

**Results:**

Econometric analysis shows that PBF led to increased job satisfaction and decreased attrition on a subset of measures, with little effect on motivation. The C1 group also experienced some positive effects on job satisfaction. The null results of the quantitative assessment of motivation cohere with those of the qualitative assessment, which revealed that workers remain motivated by their dedication to the profession and to provide health care to the community rather than by financial incentives. The qualitative evidence also provides two explanations for higher overall job satisfaction in the C1 than in the PBF group: better working conditions and more effective supervision from the District Medical Office. The PBF group had higher satisfaction with compensation than both control groups because they have higher compensation and financial autonomy, which was intended to be part of the PBF intervention. While PBF could not address all the reasons for attrition, it did lower turnover because those health centers were staffed with qualified personnel and the personnel had role clarity.

**Conclusions:**

In Zambia, the implementation of PBF schemes brought about a significant increase in job satisfaction and a decrease in attrition, but had no significant effect on motivation. Enhanced health financing also increased stated job satisfaction.

**Electronic supplementary material:**

The online version of this article (doi:10.1186/s12960-017-0179-2) contains supplementary material, which is available to authorized users.

## Background

Progress towards improving maternal and child health (MCH) outcomes requires a certain level of human resources to deliver health care services, but this has been difficult in Zambia due to a human resources for health (HRH) crisis [1–3]. Zambia faces severe health worker shortages across all levels of health care, with 93 total clinical health care workers (HCW)^1^ per 100,000 population ratio in 2009 [1]. This translates to a 60% gap in the required versus actual number of clinical health workers nationwide [4]. An average annual attrition rate of 4% from 2007 to 2009 effectively cancelled out gains made in the number of employees recruited [5]. HCWs are not evenly distributed between rural and urban parts of the country: 159 clinical health care workers per 100,000 population in urban areas versus 70 clinical health care workers per 100,000 in rural areas of the country [1]. The HRH shortage has been exacerbated by high levels of absenteeism (21%), tardiness (43%), dissatisfaction (44%), and vacancy (33.5%) rates in 2006 [6]. This situation is compounded by an imbalance in skill-mix among HCWs, and limited funding and training institutions. The implementation of HRH Strategic Plans 2006–2010 and Zambian Health Workers Retention Scheme resulted in increased staff recruitment, appointment, and retention. However, low salaries and poor working conditions continued to affect health workers’ morale. Workforce maldistribution is further exacerbated by brain drain^2^ and by increased demands placed on the health systems by patients with communicable and non-communicable diseases alike [7, 8].

From 2012 to 2014, the Zambian government introduced a large-scale performance-based financing (PBF) program to enhance the performance of existing health workers for MCH services. PBF programs typically use incentives to encourage providers to increase the provision of services and adopt best practices for quality by following explicit protocols and complying with a system of inspection and auditing.^3^ Monetary or non-monetary incentives can be directed at individual health care workers or at the health facility as a whole, and therefore districts in Zambia were randomly assigned to one of three study groups: intervention districts (PBF), input-based financing districts (C1), and pure control districts (C2).^4^ To date there is more evidence focused on the impact of PBF on patient outcomes [9–14] rather than on health care provider outcomes [15, 16]. This paper evaluates the effects of this PBF program on health workers’ satisfaction, motivation, and attrition, and examines the potential causal pathways leading to such effects.

### Theoretical framework

**Fig. 1:**
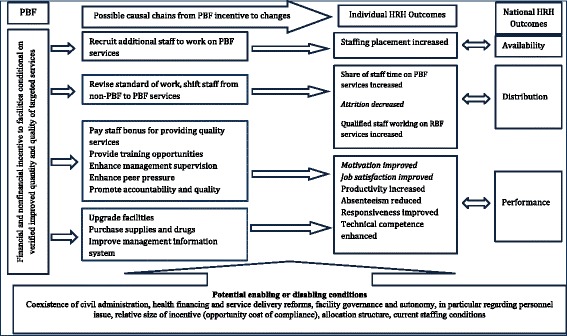
A general conceptual framework on the effects of PBF on HRH

HRH is an important node in the causal pathway from PBF to desired service provision and ultimately population health outcomes. Figure 1 is a display of our theory of change which posits that HRH—at the individual or national level—changes as a result of the implementation of a PBF program in Zambia.^5^ At the individual worker level, our model teases apart the type of incentives, as well as the combination of incentives, that could improve personnel shortage and low morale. At the national workforce level, we lay out a set of enabling and disabling conditions that are mediators of PBF and HRH. Introducing monetary incentives to designated health facilities could, in theory, help achieve systemic objectives to increase the availability, distribution, and performance of the workforce.

In this study we are interested in the differential effects of monetary incentives tied to the activities or efforts of workers (i.e., PBF bonuses) versus alternative financing modes’ (i.e., enhanced financing, status quo) effects on two individual HRH outcomes determining national workforce performance (motivation, job satisfaction) and an individual HRH outcome (attrition) determining national workforce distribution, as shown in Fig. 1. Motivation is individuals’ willingness to sustain efforts towards achieving pre-determined goals. Incentives can be a source of motivation because an individual or an organization would perform an action in order to attain a valued resource [17]. But health workers may burnout from increased demands on them to meet PBF targets in the long run. The empirical evidence of incentives on motivation is mixed,^6^ though more recent empirical evidence from sub-Saharan Africa suggests that we would expect to see a spike in motivation early in the PBF program’s implementation.^7^ We therefore hypothesize that PBF will have a positive effect on Zambian health workers’ motivation during our study period. We further hypothesize that enhanced financing would also have a positive effect on motivation, but with a lower magnitude than PBF because enhanced financing is targeted towards the health facility as a whole rather than individual health workers and is not linked directly to performance. Similar hypotheses can be made for job satisfaction.^8^

PBF is related to motivation and job satisfaction, which are predictive of turnover. The first two steps in Mobley’s heuristic model of employee withdrawal decision process is evaluation of existing job and experienced job satisfaction or dissatisfaction [18]. Psychology studies conducted since have found that job satisfaction was predicted by the reward and cost values of the job [19], and that job satisfaction was correlated with job turnover [20].^9^ High levels of motivation, like job satisfaction, reduced the risk of low- and middle-income country (LMIC) health workers’ intent of leaving their jobs [21]. The motivation-turnover relationship is mediated by affective commitment [22] and moderated by burnout [23]. These empirical studies that are conducted either in the lab or in the field cumulatively suggest that individual tenure in health care organizations is influenced by extrinsic motivation, and mediated by job satisfaction with their work setting. We thus hypothesize that PBF and enhanced financing would each have a negative effect on turnover, but enhanced financing would have a lower effect magnitude than PBF because satisfaction with compensation is a bigger determinant of turnover than other aspects of job satisfaction.

The hypothesized magnitude and direction of PBF influence on HRH are summarized in Table 1.^10^ These hypotheses reflect the expectations given the organization behavior literature and features of Zambia’s three-arm PBF design.

**Table 1:** Hypothesized magnitude and direction of PBF on HRH

	Intervention (PBF) group	Control 1, or enhanced financing not conditioned on outputs	Control 2, or “business-as-usual”
Motivation	++	+	
Job satisfaction	++	+	
Attrition	−	−	

There is a greater magnitude of effect for the intervention group than control 1 group, but the direction should remain the same. Control 2 cells are left blank because no changes are expected to occur

## Methods

The study setting (i.e., Zambia) and study intervention (i.e., PBF in Zambia) are described in Additional file 1. We gathered quantitative and qualitative data from health workers and related health centers for this study. The procedures for linking findings from qualitative and quantitative research and bringing out their complementarities can be manifold [24, 25]. Therefore, we chose to carefully interpret and triangulate the qualitative with the quantitative data because our aim is model (i.e., Fig. 1) testing [26].

### Study design

This study is part of a broader impact evaluation study aimed at measuring the effects of PBF on MCH and other health system outcomes. The evaluation follows a quasi-experimental design: 30 districts in the country were triplet-matched on key health systems and outcome indicators and randomly allocated to each study arm. Thus there are 10 PBF, 10 C1, and 10 C2 districts. The district selection process, the resulting list of districts, their health facilities, and population under study are further described in Additional file 2.

Health centers in targeted pilot districts were eligible for PBF if it employed at least one qualified health worker by the end of the first quarter of 2012. Those health centers received PBF incentive payments and emergency obstetric care (EmOC) equipment. This PBF agreement is reinforced with an institution-level contract (and a business plan) signed by DMOs and health centers, and an individual-level “motivation contract” signed by health workers and their affiliated health center. The proportion of the individual PBF staff bonus to the individual government salary was on average 10% during the entire duration of the project [27].^11^ The determination of health center payment and individual performance bonuses is further described in Additional file 3.

Health centers in the C1 group received additional financing intended to equal to the average RBF incentive payments in intervention districts, as well as EmOC equipment. This additional financing was not tied to performance, so health centers spent it as meal allowances or on rehabilitation of the health center, drugs, outreach activities, and equipment. Due to administrative bottlenecks in the financing and procurement processes adopted by the C1 districts, health facilities in the C1 group received on average a financing amount equal to 56% of the PBF group by the end of the study period.^12^ Health centers in C2 group represent “business-as-usual” since they received neither additional financing nor EmOC equipment.

This study was supported by the MOH of Zambia. The research protocol was approved by the Institutional Ethics Committee of the University of Zambia. Written informed consent was collected from all respondents. We kept all personal information confidential, and no names were used in the resulting report or journal articles.

### Quantitative data collection and analysis

Quantitative measures pertaining to the HRH outcomes of interest were assessed through surveys fielded in health centers at baseline (October–November 2011) and towards the end of the PBF pilot project (September–November 2014).^13^ A total of 186 health centers were surveyed, consisting of 86 in the PBF group, 49 in C1 group, and 51 in C2 group. Up to two health workers providing MCH services on the day of visit were interviewed for the survey in every facility, for a total of 683 staff personnel interviewed in two rounds. Statistical power for the overall evaluation was calculated using population coverage of services as key outcomes for an impact evaluation of PBF in Zambia, but power was not calculated for HRH outcomes in this study.

Motivation and job satisfaction are derived from the individual worker questionnaire and attrition is based on the facility assessment. The questions for the motivation and satisfaction were based on two existing, validated tools: Minnesota Satisfaction Questionnaire [28] and Job Satisfaction Survey [29]. In addition, the variables on well-being were derived from the WHO Well-Being Index [30]. The development of the motivation and job satisfaction constructs are described in more details in Additional file 4. Attrition was assessed by the number of authorized staff reported to have left a health center in the previous 12 months in a health facility survey.

The effects of PBF on key outcome variables were estimated with difference-in-difference framework among the PBF, C1, and C2 arms for two rounds of data (baseline and endline). Facility fixed effects analysis was performed with standard errors clustered at a district level. District grouping was taken in to account in the analysis through stratification controls. The difference-in-difference model can be summarized in the form of a linear regression equation as follows:

$$ {Y}_{ijtd}={\gamma}_0 + {\gamma}_1{\mathrm{PBF}}_d+{\gamma}_2{\mathrm{Period}}_t+{\gamma}_3\ {\left(\mathrm{PBF}*\mathrm{Period}\right)}_{d t}+{\mathrm{DP}}_d + {X}_{ijtd} + {\varepsilon}_{ijtd} $$

where *Y* is the outcome for health worker *i* under facility *j* at time *t* for district *d*; *γ*_0_ is a constant; PBF is a binary variable taking the value of 1 for districts in the PBF treatment area and 0 otherwise; Period is a binary variable where it is 1 for the post-intervention period and 0 otherwise; *γ*_1_ and *γ*_2_ are the coefficients for treatment and period, respectively; the interaction term is *γ*_3_ which indicates the difference-in-difference treatment effect; DP represents the district grouping stratification with a vector of dummy variables indicating district inclusion in particular province-level strata; *X* is a vector of worker level covariates (age, gender, and staff position); *ε* is the random error term. For most of the analysis, pairwise comparisons are separately estimated with PBF estimated with the C1 group as the default category, and then PBF with C2 as the default. The model comparing C1 with C2 groups is specified exactly the same except that PBF variable is replaced with a binary variable denoting C1. All statistical analyses were done with STATA version 13.

Results of the three-group comparisons are shown in Table 2 while results of the two-group comparisons are shown in Additional file 5. One-way ANOVA shows that at baseline there was no statistical difference among the three groups, indicating baseline balance in key characteristics that may mediate the impact of PBF on satisfaction, motivation, and attrition.

**Table 2:** Mean statistics of workers’ characteristics at baseline and endline in three groups (*N* = 683)

Variable	Baseline			Endline		
	Intervention (*n* = 147)	Control 1 (*n* = 87)	Control 2 (*n* = 92)	Intervention (*n* = 166)	Control 1 (*n* = 92)	Control 2 (*n* = 99)
Female	0.42	0.38	0.42	0.41	0.36	0.49
Education-primary	0.06	0.08	0.05	0.04	0.01	0.05
Education-secondary	0.40	0.40	0.30	***0.35***	***0.49***	***0.27***
Education-college	0.52	0.49	0.63	**0.60**	**0.49**	**0.68**
Clinical officer	0.03	0.02	0.04	0.06	0.04	0.03
Nurse	0.25	0.26	0.25	0.33	0.35	0.45
Midwife	0.11	0.13	0.14	0.12	0.09	0.15
Environmental health technicians (EHTs)	0.15	0.09	0.16	0.13	0.08	0.10
Classified daily employees (CDEs)	0.33	0.41	0.32	*0.31*	*0.38*	*0.22*
Other staff	0.67	0.59	0.69	*0.69*	*0.62*	*0.78*
Age	37.43	38.01	36.21	*35.82*	*38.51*	*35.49*
Work-absence	1.20	1.44	1.59	1.12	1.10	1.74
Work-days	5.82	6.26	6.13	6.00	6.24	6.27
Work-hours	51.45	55.90	54.55	52.07	50.33	49.61
Supervision frequency from previous year	4.52	4.32	6.65	5.62	4.58	4.54
Work experience-total	10.06	11.04	9.76	8.03	9.03	7.95
Work experience-current facility	4.55	5.40	4.39	4.27	4.67	5.09

ANOVA test of balance among three groups was performed separately for baseline and endline. Statistical significance is denoted by bold italic (*p* < 0.01); bold (*p* < 0.05); italic (*p* < 0.1)

### Qualitative data collection and analysis

The second objective of our study is to understand the possible channels through which financial incentives affect health care providers. The second objective is pursued through in-depth interviews conducted in health centers, District Medical Offices (DMOs), and provincial headquarter offices. Interviews were conducted at the beginning of PBF implementation (“baseline”; November 2011–March 2012) and three years following it (“endline”; January 2015).^14^ Organization leaders were interviewed individually, whereas staff members in a similar level on the organization chart were interviewed in a group. The sampling goal is to reach theoretical saturation, during which all major concepts are identified and additional interviews reveal no new information. A total of 81 interviews were conducted at baseline and 54 interviews were conducted at endline. The interviewees’ demographic information for baseline and endline is shown in Table 3. F4 software was used for transcription, and NVivo 10 software (QSR International Pty Ltd, Australia) was used for thematic analysis.

**Table 3:** Interviewee characteristics of the qualitative sample

Facility	Baseline	Endline
Assignment		
RBF	23 (30%)	32 (59%)
Control 1	23 (30%)	13 (24%)
Control 2	30 (40%)	9 (17%)
Type		
District Community Medical Office (DCMO)	10 (13%)	12 (22%)
Health center	66 (87%)	42 (78%)
Worker		
DCMO		
District community medical officer	2 (20%)	2 (17%)
Nursing officer	2 (20%)	2 (17%)
Human resource officer	3 (30%)	3 (25%)
Others (planner, information officer, EHT)	3 (30%)	5 (40%)
Health center		
Clinical officer	3 (5%)	2 (5%)
Registered nurse	2 (3%)	1 (2%)
Enrolled midwife	15 (23%)	4 (10%)
Enrolled nurse	15 (23%)	5 (12%)
Environmental health technician (EHT)	17 (26%)	7 (17%)
Classified daily employee (CDE)	12 (18%)	7 (17%)
Lab technician	2 (3%)	16 (28%)
Gender		
Male	42 (55%)	27 (50%)
Female	34 (45%)	27 (50%)
Highest academic/professional qualification		
Degree	3 (4%)	3 (6%)
Diploma	29 (38%)	18 (33%)
Certificate	32 (42%)	17 (31%)
Senior secondary education	2 (3%)	9 (17%)
Junior secondary education	10 (13%)	7 (13%)
Total	76 (100%)	54 (100%)
Job experience (in years)		
Mean (*n*; standard deviation)	10.6 (76; 9.2)	9.8 (54; 8.7)
Number of years working in district		
Mean (*n*; standard deviation)	8.2 (76; 8)	7.9 (54; 6.9)
Number of years working in a health facility		
Mean (*n*; standard deviation)	5.2 (76; 4)	4.9 (54; 5.1)

## Results

**Fig. 2:**
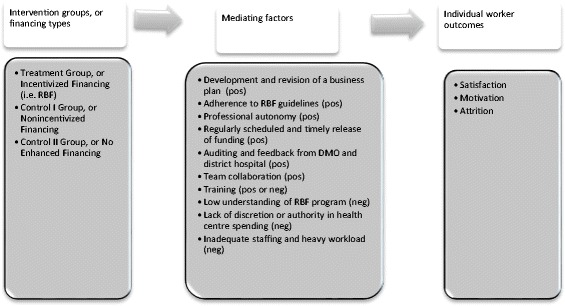
Mediators of PBF and HRH

In this section, we present results for the three HRH dimensions (motivation, job satisfaction, attrition), study group differences for each dimension’s general construct scores, and for each construct’s constituent variables. Figure 2 summarizes the intermediary factors that emerged from interviews, which we will explain along with the regression analysis results.

### Motivation

We did not find support for our hypotheses for any of the eight motivation constructs with one exception: respondents in the PBF group reported, out of 100%, 2.42% (*p* < 0.1) higher on the personal well-being scale between baseline and endline than those in the C2 group (Table 4). This aggregate finding is driven by respondents in the PBF group who felt more calm and relaxed in the 2 weeks prior to reporting between baseline and endline than those in the C1 group (9.48% higher; *p* < 0.1) or those in the C2 group (5.69% higher; *p* < 0.05). The group differences for the eight motivation constructs are summarized in Table 4.

**Table 4:** Estimated effect of PBF and enhanced financing on motivation

	Intervention v. control 1 (*N* = 448)	Intervention v. control 2 (*N* = 462)	Control 1 v. control 2 (*N* = 345)
	*β* (standard error)	*β* (standard error)	*β* (standard error)
Teamwork	0.39 (3.13)	0.93 (1.43)	1.62 (3.51)
Autonomy	0.82 (4.31)	1.31 (1.77)	1.30 (4.49)
Recognition	−0.38 (3.28)	−0.84 (1.33)	−0.89 (2.85)
Change	−2.10 (2.66)	1.03 (1.24)	3.83 (2.64)
Self concept	−0.73 (1.87)	0.77 (1.08)	2.21 (2.36)
Work environment	−1.79 (2.60)	1.26 (1.26)	4.31 (3.03)
Leadership	−3.08 (4.89)	1.21 (2.61)	5.55 (5.15)
Well-being	1.10 (2.98)	2.42* (1.24)	3.93 (2.50)

Coefficients, standard errors, and *p* values are for the interaction between the random assignment (intervention, control 1, control 2) and study period (baseline, endline). They are obtained from pair-wise regressions—facility fixed effect models controlling for workers’ characteristics. Robust standard errors are clustered at the district level

*p < 0.1

Looking specifically at the individual questions under each motivation construct (Additional file 6), the PBF appears to have encouraged staff to willingly give their time and help each other out when someone fell behind or had difficulties with his or her work; 3.77% higher (*p* < 0.05) between baseline and endline for the PBF than for the C2 group. Finally, three of the motivation questions seemed to discern group differences, which could be used and elucidated in future PBF research. The three questions are as follows: “I would prefer to work somewhere else than in this facility” (12.27% lower between baseline and endline for the PBF group than for the C1 group; *p* < 0.1); “My facility is a very dynamic and innovative place. People are willing to take risks to do a job well done” (5.06% higher between baseline and endline for the PBF group than for the C2 group; *p* < 0.1); and “Following procedures and rules is very important in my facility” is 1.95% (*p* < 0.1) and 4.26% (*p* < 0.1) higher between baseline and endline for the PBF and C1 group when each of them was compared with the C2 group.

Interviews, in accordance with the null effects of PBF on motivation, revealed that remuneration alone could not adequately address two causes of de-motivation: high workload and low staffing levels. Financial bonuses paid out by the PBF program were adjusted by workload, but they were not directly tied to the staffing level. This became problematic in health care settings where the workload rose but staffing levels remained the same:

There is a small clinic…with [urban clinics having] more nurses than we have here. We have tried to tell them, ‘you guys give us some more nurses because we are over worked here’. However, they behave as if there is more work there than there is here. —Nurse, Gwembe (PBF group)

Rural health workers felt they worked longer hours compared to their urban counterparts who worked in shifts because urban clinics were better staffed. As a result, they were not able to get any days off, rest after completing work, or have some time for personal responsibilities. High workload, exacerbated by chronic staff shortages, ultimately compromised health care workers’ motivation to provide high-quality services.

Respondents provided reasons other than monetary incentives for remaining in the health workforce during the interviews, namely professional training and obligation to serve patients:

First of all you have to be proud about your own profession and if you leave it who will handle it? … [If] I leave my job because of the small salary and whatever. I think that’s not right. I am just happy to be what I am as a doctor. The profession itself is a motivating factor. —Provincial Medical Officer, Livingstone (not in a study group)

Even when it is over our working hours, we still come to the clinic and attend to our patients because we would not know what would happen; maybe the patient’s condition can be worsen if we do not attend to them early enough. —CDE, Itezhi-Tezhi (C1 group)

Our results thus raise important secondary questions regarding the influence of extrinsic motivators (e.g., bonuses from PBF scheme) versus that of intrinsic motivators (e.g., workers’ internal desire) for serving patients and the community at large.^15^ We find Zambian health workers’ performance appears to have been driven more by internal rewards, such as passion for their job or professional integrity, and are largely unaffected by the PBF bonuses or enhanced financing.^16^ We echo Judson et al. point that it is complex to determine the right balance of extrinsic and intrinsic motivators in order to achieve the “value goal” in PBF schemes [31].

### Job satisfaction

We found support for hypotheses with overall job satisfaction and compensation (see Table 5).

**Table 5:** Estimated effect of PBF and enhanced financing on job satisfaction

	Intervention v. control 1 (*N* = 448)	Intervention v. control 2 (*N* = 462)	Control 1 v. control 2 (*N* = 345)
	*β* (standard error)	*β* (standard error)	*β* (standard error)
Relationship outside facility	1.64 (2.96)	0.43 (1.49)	−0.59 (3.12)
Relationship within facility	−4.16 (2.82)	0.48 (1.02)	4.94* (2.59)
Work conditions	6.39 (5.12)	4.37* (2.18)	2.20 (5.90)
Recognition	1.44 (2.84)	0.09 (1.32)	−1.44 (2.24)
Opportunities	4.69 (4.18)	3.64* (2.00)	2.30 (5.24)
Compensation	8.64** (4.08)	3.88* (1.99)	−0.82 (4.87)
Overall satisfaction	−0.48 (3.96)	4.75** (2.14)	10.31** (3.94)

Coefficients, standard errors, and *p* values are for the interaction between the random assignment (intervention, control 1, control 2) and study period (baseline, endline). They are obtained from pair-wise regressions—facility fixed effect models controlling for workers’ characteristics. Robust standard errors are clustered at the district level

*p < 0.1; **p < 0.05

More specifically, we estimated a statistically significant increase of 4.75% (*p* < 0.05) in overall job satisfaction between baseline and endline for the PBF versus C2 group. The same effect was more pronounced—10.31% (*p* < 0.05) higher—between baseline and endline for C1 versus C2 group. Though not statistically significant, there is a 0.48% lower overall job satisfaction between baseline and endline for the PBF than C1 group. Overall job satisfaction was found in the following order, from highest to lowest: enhanced financing, PBF, and pure control. This order for overall job satisfaction is contrary to what we hypothesized and contrary to the order we found for satisfaction with compensation.

For compensation, respondents in the C1 group reported an average of 8.64% (*p* < 0.05) lower between baseline and endline for being rewarded for their hard work than their counterparts in the PBF group. Likewise, respondents in the C2 group reported 3.88% (*p* < 0.1) lower points between baseline and endline for compensation than those in the C1 group. There was not a statistically significant difference between the C1 and C2 groups. As expected, we observed higher average satisfaction with compensation in the PBF group than in the C1 group, followed by C2 group. Full results for all questions under each satisfaction construct are in Additional file 7.

The PBF program added monetary incentives whereas enhanced financing provided material resources to improve health infrastructure, both of which increased workers’ job satisfaction. PBF and, to a lesser degree, enhanced financing groups both had a consistently positive effect on satisfaction with working conditions. Contrary to what we hypothesize though, C1 was not statistically different than either the PBF or the C2 group in terms of satisfaction with work conditions. Those in the PBF group had increased satisfaction over availability of supplies between baseline and endline relative to those working in health centers that received enhanced financing (12.97% higher; *p* < 0.1) or to those who did not receive additional financing (7.73% higher; *p* < 0.05). This finding is surprising given that the PBF and C1 groups received the same EmOC equipment, and C1 additionally received financing not tied to performance. But it makes sense in light of an unintended effect revealed in the interviews: staff workers used personal PBF bonuses as “re-investment funds” to improve their own working environment, as is the case in Gwembe.

PBF respondents reported lower overall job satisfaction than enhanced financing respondents for two reasons. First, health care workers felt improving working conditions was more important than focusing on monetary incentives. The shortages and/or lack of infrastructure and equipment were major barriers to the delivery of quality health services:

When I lose a client [dies] as a result of a situation about which I could have been able to do something but there is nothing to use, that de-motivates. It’s better if a client dies from malaria in a situation where you were able to give him/her quinine or Coartem. Not where you are supposed to prescribe the drugs and they are not there; that is really de-motivating. —Nurse, Gwembe (PBF group)

The availability of transport was also reported as important for job satisfaction given that many of the rural health centers were hard to reach. Motorbikes enabled health workers to travel to outlying areas to conduct their outreach activities.

Second, the high frequency of administrative audits and quality assessments tied to the PBF program also affected overall job satisfaction. All health centers in Zambia are supposed to receive supervision and support in the form of an administrative audit from the DMO once a month and a quality assessment from the hospital once a quarter, but we found that the C1 and C2 groups received less frequent visits than the PBF group.^17^ The audits and assessments had their drawbacks for the PBF group. Respondents felt that the visits happened too frequently for any observable difference to be observed. Further, some respondents complained that the verification teams would visit unannounced (intended to prevention falsification of results) when the staff were inundated with work, when the health center is short of staff, or when some staff conversant with PBF issues and processes were working outside the health center. Finally, some of the health centers resented the DMO meddling with their internal affairs:

We were told by the DMO we could include allowances [in the business plan] only with an authority letter from PBF. So when you look at it …strictly speaking, autonomy was not there. —Nurse, Isoka (PBF group)

This nurse received the DMO’s guidance as a directive. Countering this, respondents representing the DMO felt that some health centers spent money outside the parameters of their business plans, and therefore they had to be corrected.

Nonetheless, PBF group had more autonomy over the allocation of resources than either one of the control groups, by design.^18^ They had access to their account balance, and could therefore plan ahead:

For the percentage that was there under PBF; it was not for the DMO to plan for us. When we got that money; 25^19^ percent of that money was for the center to plan what to buy since we knew the things that we did not have. This has been a plus, because we were able to buy things on our own. —Midwife, Gwembe (PBF group)

PBF funds provided this respondent with a sense of security because health center staff members were given spending discretion, but they still had to disburse the funds in a timely manner. If there was a delay in disbursing government funds, as was the case in Chipepo and Gwembe, health centers have had to use part of their PBF funds to cover activities outside the scope of their original business plan.

The C1 group had less financial autonomy than the PBF group. Health centers in the C1 group still followed the traditional protocol of determining their internal needs, then submitting their purchasing requests to the local DMO for approval:

We don’t directly receive that [equivalent of PBF amount of] money for us to buy our stuff. The district buys for us…I think that the person receiving it on the other end [in DMO] would not see the importance and may just leave it out. Therefore, we should have been receiving that money directly ourselves; since we are the ones working here and we are the ones who know what we need and what we don’t need. —Staff, Itezhi-Tezhi (C1 group)

This respondent simply did not think the Itezhi-Tezhi DMO, acting as a middleman between the MOH and the health center, honored his or her health center’s needs and the needs of the wider community. In sum, our qualitative assessment revealed individual job satisfaction and the relationship between health centers and DMOs were both affected by the amount of available resources, PBF program-related assessments, and autonomy to control financial resources. On net it appears that overall satisfaction was most elevated in the C1 group, followed closely behind by the PBF group.

### Worker attrition

As shown in Table 6, the coefficients in the comparison between PBF and either C1 or C2 all have the expected negative signs, interpreted as less staff leaving the PBF health centers than the ones in the two control groups. However, only two professional categories were statistically significant. There were 0.10 (*p* < 0.05) fewer administrators on average who left health centers in the PBF group than in the C1 group. The PBF intervention group also had lower turnover of nurses, with 0.14 (*p* < 0.05) fewer nurses on average who left when compared with the C2 group.

**Table 6:** Estimated effect of PBF and enhanced financing on attrition

	Intervention v. control 1 (*n* = 448)	Intervention v. control 2 (*n* = 462)	Control 1 v. control 2 (*n* = 345)
	*β* (standard error)	*β* (standard error)	*β* (standard error)
All staff	−0.03 (0.05)	−0.02 (0.02)	−0.00 (0.06)
Clinical officer	−0.05 (0.05)	0.04 (0.03)	0.14* (0.08)
Administrator	−0.10** (0.05)	−0.01 (0.01)	0.07 (0.05)
Nurse	−0.19 (0.15)	−0.14** (0.06)	−0.09 (0.15)

Coefficient denotes number of staff in each category who left the facility permanently in the last 12 months. Coefficients, standard errors, and *p* values are for the interaction between the random assignment (intervention, control 1, control 2) and study period (baseline, endline). They are obtained from pair-wise regressions—facility fixed effect models controlling for workers’ characteristics. Robust standard errors are clustered at the district level

*p < 0.1; **p < 0.05

Low compensation was a reason to look for another job. Health workers in rural areas further felt that they were in a disadvantaged position compared to their counterparts in urban areas because they had poor access to the media, information, and training opportunities. This is tied to other reasons for staff turnover reported such as retirement, illness, marriage, schooling for workers’ children and dependents, lack of accommodation, or the need to pursue further academic studies or professional training.

The HRH situation generally improved after the PBF program was introduced to their district:

For the past two years that we have been with this PBF, I have never heard any staff saying they want to go to the hospital…the same people are comfortable in the health centre…nobody has requested for any transfer or even talking about it. -Nurse, Isoka (PBF group)

Many health workers reported engaging in parallel income generation activities such as farming and business during the baseline interviews. This corresponds to 21% of a nationally representative sample of health workers reported being involved in income-augmenting activities in 2006 [6].^20^ But health centers became more attractive as places to work in than district hospitals due to the incentives from the PBF program.

Staff shortages and understaffing are problems strongly endorsed during the baseline interviews. Managers started paying closer attention to staffing because the allocation of bonuses through the PBF program depended heavily on the availability and placement of qualified staff members in the health center.

Sometime back, some centers used to be manned by unqualified staff but when the [PBF] program came, management was pressured to the extent that we needed to find where we could source some qualified staff, such as from the hospitals to go to the [health] centers… Things have changed now compared to the past because every health facility now has a qualified health staff but then, they are not enough. —DMO, Isoka (PBF group)

But with the emphasis on quality as in skilled personnel; that [PBF program] has helped us put every member of staff where they are supposed to be.—EHT, Gwembe (PBF group)

Having qualified co-workers and greater role clarity prevented job turnover, especially in the PBF group.

In some of the health centers, such as the three we visited in Isoka, health cadres chose to give up part of their individual bonuses to hire non-specialized yet qualified staff out of institution-wide funds. By doing so, they hoped to improve the amount of bonus points earned the following quarter and, in turn, it would pay off in higher individual bonuses in the long run. This is a double gain in that the health center is better staffed to provide quality services and it helped increase the relative size of bonus that everyone on staff can earn. Not all health centers in the PBF group were able to do the same because the bonuses they earned were inadequate due to low catchment population, low performance, lack of a midwife, etc.

## Discussion

In this mixed-methods study, we investigated whether a national government-implemented PBF scheme improved three HRH outcomes in Zambia: motivation, job satisfaction, and attrition. Our econometric estimates suggest that PBF led to increased job satisfaction for a small number of constructs and decreased attrition of administrative staff and nurses, but PBF did not lead to marked effects on motivation. We also found support for overall job satisfaction and compensation, with both PBF and enhanced financing experiencing a more positive effect compared to pure control. However, the gains were slightly lower for the PBF than for the C1 group. For attrition, we observed lower turnover for administrators in the PBF group compared to either of the control groups. Incentive schemes may not have the same effect on HRH outcomes in another national context, which differ on labor market conditions including changes in staff salaries, retirement age, transfers within and across districts, and education status [32, 33].^21^

There are indications that PBF has a minor impact on elicited motivation, which is also what Dale found for Afghanistan’s performance-based payment program [34]. One channel through which motivation is affected is when individuals feel strained because they are held accountable for outcomes not under their direct control, which we did not find either quantitative or qualitative evidence of [35]. A second concern is that a large enough financial incentive package would diminish personal reasons to work [36–39]. But our interviews revealed a general balance of both extrinsic and intrinsic sources of motivation. Some interviewees revealed self-introspection, which helped us to ascertain how people energize themselves to persist working at healthcare delivery.

Complementary to econometric results, our qualitative assessment shows positive evidence on health workers’ job satisfaction and attrition from PBF and, to some extent, from enhanced financing. The evidence suggests that in response to PBF, health care providers worked harder and some also increased community outreach activities in order to earn more bonuses. However, a higher workload as an imperative to earn points added pressure on health workers. Among PBF respondents, there was a sentiment that the district supervision visit was too frequent and sometimes too stringent, thus decreasing overall job satisfaction. Those in the PBF group did enjoy autonomy to solve their own problems, which was not there in practice and thus lessened job satisfaction with compensation for those in the control groups. Ultimately, PBF made health centers more attractive to work in than hospitals, and with more specialized and non-specialized positions filled, it allowed skilled providers to focus on caring for patients. Health workers appreciate working in a PBF health center not only because of the financial incentives, but because of professional dedication, capacity to serve the community, and opportunities for professional development.

We can envision at least four limitations of this study. The district pairing design could be somewhat compromised with “contamination” across groups.^22^ However, baseline characteristics were similar among workers across the three groups in Table 1, which lends confidence that results were not susceptible to confounding bias. Furthermore, as the PBF and C1 interventions were administered at the district level, communications between health staff were much greater within than across districts.

Second, as this is an observational study, recall bias could affect the accuracy of our estimates, especially because a recall period was not specified in the survey for the questions related to motivation and job satisfaction. However, we believe such psychometric properties would not differ for the three study groups since all of the respondents completed the same survey in the same time period. Third, study instruments could be improved to deepen the understanding of HRH and MCH outcomes. Designing the interview guide so that interviews are carried out with non-managerial staff on a one-on-one basis would minimize normative bias. Also, mediation analysis can be used in future studies to test whether, and to what extent, motivation or job satisfaction mediates the relationship between PBF and attrition or PBF and staff performance. This would not only improve upon our theory of change but, from a performance management standpoint, improve the design of systems of incentives and appraisal put in place to produce the level of performance necessary to achieve health service objectives.

Lastly, job satisfaction and motivation are abstract concepts. We expect job satisfaction and motivation to increase with additional funding, and indeed we do find evidence supporting this for job satisfaction. But staff responses to our survey are nuanced in that they are responding to different levels of pay and to the conditions of the overall health system. PBF increases demands on their job roles and pressure to meet PBF targets. This stress has an impact on worker productivity, turnover, and well-being over and above the direct extrinsic rewards of provider incentives. Therefore, future research should examine a confluence of factors related to staff responses such as employee involvement in setting PBF-related targets, ability to control the factors which affect meeting those targets, their perception of the transparency of performance evaluation process and fairness of reward process, and adequacy of program funding level. Although the fields of organization behavior, management, and industrial and organizational psychology have made progress on this topic, it is still a challenge to quantify health workers’ job satisfaction and motivation, especially in health contexts of LMICs.

## Conclusions

This study contributes to the nascent literature on the effects of PBF on health worker outcomes in LMICs. The Zambia pilot studied here conferred both financial and non-financial rewards, such as raising standards of performance, developing accurate performance measurement systems, and training managers on how to give effective feedback. Enhanced financing, which was used to improve work conditions, can also encourage health personnel to work harder and stay in rural communities. Our study calls for a careful examination of the contextual factors which form the sufficient conditions to make the desirable effects of performance-based or enhanced financing manifest. While some of the conditions are beyond the immediate Zambian program implementers’ span of control, such as staffing shortages, many are under their purview, such as the quality of supervision, communication, and refresher trainings for staff.

The adoption of PBF is part of health system reforms [40, 41]. The research literature has not explicitly focused on an important mediator between PBF incentives and desired health services outcomes: HRH. The Zambia PBF program offered incentives to achieve desired MCH outcomes and, in the process, modified individual health care provider behavior and investments in entire health centers. We drew the link between PBF and three HRH outcomes because poor job satisfaction and motivation lead to poor performance and higher attrition, thus disrupting continuity of care for patients and, in aggregate, incurring higher costs for the health system. Our study not only highlights effective and sustainable ways to strengthen the health workforce in Zambia, but it has implications on how to strengthen HRH’s relationship with other health system building blocks in LMICS.

## Additional files

Additional file 1: Description of Zambia and PBF in Zambia [1, 27, 42–52]. (DOCX 150 kb)
Additional file 2: District selection of Zambia’s PBF Program. (DOCX 41 kb)
Additional file 3: Determination of performance bonuses to individual staff members of health centers. (DOCX 139 kb)
Additional file 4: Description of motivation and job satisfaction constructs and variables [53]. (DOCX 135 kb)
Additional file 5: Mean statistics of health worker characteristics at baseline and endline, two-group comparisons. (DOCX 89 kb)
Additional file 6: Regression results for motivation variables. (DOCX 82 kb)
Additional file 7: Regression results for job satisfaction variables. (DOCX 83 kb)

## Abbreviations

ANC: Antenatal care
CDE: Classified daily employees
CET: Cognitive Evaluation Theory
CSO: Central Statistical Office
DMO: District Medical Office
EmOC: Emergency obstetric care
HMIS: Health Information Management System
HRH: Human Resources for Health
LMICs: Low- and middle-income countries
MCH: Maternal and child health
MOH: Ministry of Health
P4P: Pay For Performance
PBF: Performance-based financing
PMO: Provincial Medical Office
PNC: Postnatal care

## Footnotes

1 Clinical health worker encompasses 12 categories: clinical officers, dental surgeons, doctors, nutritionists, lab scientists, pharmacists, physical therapists, radiographists, midwives, nurses, environmental health personnel, and oral health. Clinical health workers are not the same as administrators, who also work in health facilities.

2 Poor staff morale and weak incentives lead to emigration or migration, which in turn result in a health worker shortage [54, 55]. An initial wave of Zambian health workers migrated to countries in sub-Saharan Africa, such as South Africa, Botswana, and Namibia [56]. Subsequent waves migrated to Europe, North America, Australia, and New Zealand. An exodus of health professionals has also been observed within Zambia from rural to urban areas, from the public to the private sector, and from curative to preventive care [57, 58].

3 PBF is a strategy to address inadequate performance-reward linkages and, more generally, health system reform [40, 59]. In this paper we adopt Soeter et al.’s definition of PBF as “fee-for-service conditional on quality of care” [60].

4 Monetary incentives (i.e., bonus payment) can either be awarded for achievement of predefined performance targets or withheld if targets are not reached. Individuals and organizations may also respond to non-monetary incentives of PBF schemes such as enhanced supervision, data system development, and institutional rankings [61, 62].

5 A wide array of moderators on the “meso-levels,” or intermediary levels, that could weigh on PBF’s influence on worker outcomes, which we acknowledge them though not account for them explicitly in this study. Beyond concerns over PBF implementation is fungibility, i.e., whether health managers allocate revenue to health worker bonuses or to other assets of equivalent value, say, equipment, supplies, and capital improvements to the health care facility. Toonen et al. considered autonomy, management capacity, and an understanding of PBF concepts to be important for implementing PBF programs in sub-Saharan Africa’s health sector [63]. Mohammed et al. urged a closer look at the “know–do” gap, or the gap between what health workers know how to do—and actually do (Mohammed RL, Herbst C, Leonard K, Goldberg J.: Crossing the Three-Gap Divide with PBF, unpublished). PBF interventions can reduce this gap through improved accountability and supervision structures, and more generally via an improved working environment.

6 Eisenberger et al. argued that rewards could have a positive, negative or null effect on motivation depending on the nature of the task assigned in controlled laboratory settings [64]. Woolhandler et al. further questioned the causal direction posed by rewards on motivation in health care settings in high-income countries [65].

7 We make this assertion following Bhatnagar and George’s observations in Nigeria [66] and Kalk et al.’s observations in Rwanda [67].

8 Job satisfaction is workers’ personal satisfaction relative to their work situation [68]. The correlation between income and subjective well-being has been observed both within and across countries [69]. Yet job satisfaction is a multi-faceted concept. Heneman et al.’s results indicated a positive relationship between pay-for-performance perceptions and pay satisfaction [70]. Judge et al.’s meta analysis results suggested that pay level is only marginally related to job satisfaction [71]. Job satisfaction is not just about job conditions but also personality. In an earlier study, Judge et al. found that traits of core self-evaluations, or positive self-concept, are positive dispositional predictors of job satisfaction [72]. The PBF scheme in Zambia primarily responds workers’ satisfaction with their compensation, but it might also respond to the other six aspects of job satisfaction we measure. Therefore, like motivation, we hypothesize that PBF has a positive effect on job satisfaction. We further hypothesize that enhanced financing would also have a positive effect on job satisfaction, but with a lower magnitude than PBF because enhancing financing primarily addresses one aspect of job satisfaction, or satisfaction with work conditions.

9 Porter et al.’s longitudinal study results further suggested that satisfaction with pay is most acute at time points closest to when psychiatric technicians intend to leave their organization [73].

10 We expect to see stronger effects on HRH results for the intervention (PBF) group than for the control 1 (C1; enhanced financing) group, but the direction is expected to remain the same for the following reason: RBF can be interpreted by health workers as a reward for their individual efforts, whereas enhanced financing can be interpreted by them as a signal of recognition for collective efforts through improvements on working conditions. Both incentive schemes are expected have positive, knock-on effects on HRH outcomes compared with the control 2 (C2; pure control) group.

11 The amount of bonus received by each staff member was dependent on a number of factors: individual performance scores taken during a performance appraisal, actual PBF income made, investment priorities, the number and composition of staff at the health center, and individual government salary levels. The ratio was higher at the start of the PBF project but dropped after about 6 months of the PBF project due to an increase in government salaries ranging from 100 to 200% [27]. Nonetheless, PBF staff bonuses led to an absolute increase in the personal income for staff but by different margins/percentages across staff and health centers.

12 It should also be noted that, unlike the PBF districts, health centers in C1 districts did not have devolved autonomy, enhanced supervision, training, access to PBF reference materials, and data monitoring and verification that were also part of the PBF intervention.

13 The surveys collected information on human resources and physical capacity, facility governance, practitioner knowledge, outreach activities and other initiatives, and quality of care and practitioner behavior through patient exit interviews.

14 At baseline, health centers and DMO offices in Gwembe (PBF group), Itezhi-Tezhi (C1 group) and Mazabuka (C2 group) in the Southern Province of Zambia were included in the sample. Southern Province reflects the median levels of socio-economic status and health indicators across all the ten Zambian rural provinces, so focusing data collection efforts on this province enabled easier identification of cross-cutting issues for PBF and human resources. Five additional interviews with conducted with Provincial Medical Officers in Livingstone. At endline, health centers in four districts (Isoka, Gwembe, Itezhi-Tezhi, Mazabuka) were purposely sampled based on a multitude of characteristics such as remoteness, size of catchment area, urban/rural split, performance, and staff-mix.

15 Cognitive evaluation theory (CET) predicts that extrinsic rewards would diminish, if not displace, intrinsic interest [36]. Deci et al.’s meta analysis concluded that extrinsic awards decreased intrinsic motivation regardless of the form of incentive: engagement-, completion-, or performance-contingent awards [39]. Decreased intrinsic motivation negatively affects worker autonomy, purpose, altruism and competence [74]. Huillery and Seban found lower staff attendance and on-the-job effort after the PBF pilot ended in the Democratic Republic of Congo, which they attribute to not only lowered motivation, but a switch from intrinsic to extrinsic motivation on the workers’ behalf [75]. However we do not find evidence in support of CET.

16 Likewise, Brock et al. have found that peer scrutiny and encouragement alone can determine quality of care provided by clinicians in their sample [76].

17 During administrative audit and quality assessment visits, the verifiers would look at all aspects of PBF program implementation and see how health centers were performing against standards and benchmarks. After their visit, the verifiers would debrief health center staff on their strengths and weaknesses, and with the health center cadres devise potential solutions.

18 PBF funds were determined from performance and then disbursed from the MoH directly into each health center’s bank account. PBF funds did not pass through the DMO. The health cadres of Isoka and Gwembe districts, both PBF groups, had high involvement in prioritizing needs and use of PBF money without having to wait for the DMO to tell them what to do.

19 The amount should be 40%.

20 Health staff who were engaged in income-generating activities in order to augment their salaries has also been observed in Democratic Republic of Congo [77], in Sierra Leone [78], and in Tanzania [79].

21 For example, Zambia raised salaries for all government workers in 2011 and in 2013 by between 100 to 200% which had an effect on the size of the staff bonus.

22 For example, health workers from either of the control groups may have sought employment in one of the health centers in the PBF group. Health workers across the three study groups could also have been sharing information about how to generate additional income from means other than the PBF program [80].
