# Supplementary material for: Incentives to change: effects of performance-based financing on health workers in Zambia
Source: Hum Resour Health. 2017 Feb 28;15:20. doi: 10.1186/s12960-017-0179-2 (PMC5331731; doi:10.1186/s12960-017-0179-2)
Supplement: Additional file 1: — Description of Zambia and PBF in Zambia [1, 27, 42–52]. (DOCX 150 kb) [file 12960_2017_179_MOESM1_ESM.docx]

**Additional file 1. Description of Zambia and PBF in Zambia**

## *Study setting-Zambia*

Zambia is a low-income country with a population of 15.7 million people, of which life expectancy is 60 years old and 60 % live in rural areas [49]. The 2013-14 Zambia Demographic and Health Survey shows the maternal mortality ratio (398 deaths per 100,000 live births), infant mortality rate (45 deaths per 1,000 live births), under-five mortality rate (75 deaths per 1,000 live births), and HIV prevalence (13% among adults) to be high [50]. Yet the utilization of MCH services varies: 47% for births attended by skilled health personnel, 60% of four or more antenatal care visits, and 80% of measles immunization among one-year olds [51]. Factors other than HRH that hamper progress in improving these statistics include inequalities in service provision and limited financial resources for health facilities (i.e., hospitals, health centers). The total expenditure dedicated to health—5% of GDP in 2013—is disproportionately allocated to disease-specific programs as opposed to investments in other areas which would strengthen health systems, such as, facility improvements, increased staffing, or workforce development [52].

Zambia’s health system is decentralized, with functions and authority devolved in four tiers: central Ministry of Health (MOH), provincial medical office (PMO), district medical office (DMO), and neighborhood health committee. Health service delivery facilities in Zambia differ by level and by ownership. In 2010, there were 1,882 health facilities, namely health posts (15%), rural health centers (56%), urban health centers (23%), level-1 hospitals (4%), level-2 hospitals (1%), and level-3 hospitals (0.3%) [1]. Hospitals of level 1, 2, and 3 are respectively overseen by DMOs, PMOs, and MOH. The majority of health facilities are public (79%), followed by faith-based (6%) and private (14%) ownership [1]. The health system has experimented with various interventions to improve health care delivery such as governance [53], and service planning [54] and financing policy [55].

## *Study intervention-Performance-based financing in Zambia*

The government of Zambia, with the assistance of the World Bank, began to implement a PBF pre-pilot project in Katete District in 2008 and a pilot in 10 rural districts between 2012 and 2014 with the aim to link financing to results. The PBF in Zambia was designed to adhere to four principles: to increase autonomy in health center management and planning among service providers; to increase accountability to the community by involving them in managing services; to improve reporting through the usage of management tools (i.e., business plans, contracts, external verification, investment fund) to plan for services; and to strengthen the health system by separating the functions of policy formulation, service delivery, and regulation. In general, the conditions documented as favorable for the success of PBF are numerous, including strong leadership and management support, accurate information and reporting systems, and increased funding and training [56-58].

The Zambia PBF pays the providers for service provision and quality of select high priority MCH services. The data reported by health centers are verified both in quantity by the DMO and in quality by district hospitals before payment is made. Zambia is a suitable context to test the implementation of a PBF program on workforce strengthening in these areas because rural health centers have the autonomy to plan and use the PBF revenue: up to 60% of their revenue can be shared among the staff as individual bonuses, while a minimum of 40% has to be invested in maintaining the working conditions of the facility itself [59].

The Zambia PBF pilot focused on rural areas for two reasons. Firstly, MCH status is lower in rural than urban areas. Women in rural areas are more likely to have an unmet need (24%) for family planning than women in urban areas (17%) [50]. And children in rural areas are more likely to die young, as indicated by under-5 mortality rate of 85 deaths per 1,000 live births, than children in urban areas of 72 deaths per 1,000 live births [50]. Secondly, incidence of poverty in rural areas, at 78%, is more than double that in urban areas, which is estimated at 28% [60].

This study is part of a broader impact evaluation study aimed at measuring the effects of PBF on MCH outcomes. More specifically, we focus on the effects of PBF on HRH outcomes in this paper. Our study has two main objectives: (1) to estimate the effects of a PBF intervention on three key dimensions of HRH: motivation, job satisfaction, and attrition, and (2) to understand the channels through which financial incentives -either through PBF or enhance financing- lead to the observed effects on providers. The first objective is pursued through statistical comparison of data attained from the PBF group with those from the two control groups on different HRH constructs. And the second objective is pursued through in-depth interviews conducted in health centers.
