# Supplementary material for: Incentives to change: effects of performance-based financing on health workers in Zambia
Source: Hum Resour Health. 2017 Feb 28;15:20. doi: 10.1186/s12960-017-0179-2 (PMC5331731; doi:10.1186/s12960-017-0179-2)
Supplement: Additional file 2: — District selection of Zambia’s PBF Program. (DOCX 41 kb) [file 12960_2017_179_MOESM2_ESM.docx]

**Additional file 2. District selection of Zambia’s PBF Program**

The Zambian government provided input on the sampling procedure for the evaluation of the national PBF program, the pilot of which lasted from April 2012 to October 2014. To select study districts, PBF program evaluators gathered district-level information on three indicators: district health administrative capacity, district population health services outcomes, and district population living standards. They then created indices for 74 districts in Zambia on each of the three indicators. Finally, program evaluators combined data on the three indicators through principal components analysis into one index. Within each of the ten Zambian provinces, they selected three districts at or near the provincial median index score, and then randomly assigned the selected district to one of the three study groups: PBF, C1, and C2 group. All health centers in the 30 selected districts were recruited to participate in the evaluation study.

*District health administrative capacity*: The administrative capacity of the district is measured as an index derived from principal components analysis based on the following three measures of District Health Management Team (DHMT) performance:

- The average facility level stock-out rate of key commodities over the years 2006 and 2007.
- The average supervisory visit rate from DHMT to all facilities over the years 2006 and 2007.
- The rate of under-5 population covered by immunization campaigns in 2006 and 2007.

All three of these measures reflect different aspects of DHMT capacity and are combined into an index that is then sorted into quintiles.

*District population health*: The population health indicator derives from the following three measures:

- The average in-facility delivery rate over the years 2006 and 2007.
- The average facility TT coverage rate over the years 2006 and 2007.
- The average facility post-natal coverage rates over the years 2006 and 2007.

These three indicators are then combined into one health index by principal components analysis and sorted into quintiles.

*District living standards*: The socio-economic conditions prevailing in the district reflect a range of measures taken from the 2006 Living Conditions Monitoring Survey that are combined by Kaboso and Temba (2009) into a material deprivation index.

The three separate district indicators are re-combined through principal components analysis into one index that is then sorted into quintiles. Within each province, three districts at or near the provincial median index score derived from these measures are selected and then randomly assigned to either the intervention or one of the two control statuses, following the evaluation design described in the Study Design subsection.

The districts selected for the three study groups are as follows:

| **Intervention Group - RBF Intervention Districts** | | **Control 1 Group—Enhanced Financing** | | **Control 2 Group—“Business as usual”** | |
| --- | --- | --- | --- | --- | --- |
| *Province* | *District* | *Province* | *District* | *Province* | *District* |
| Central | Mumbwa | Central | Kapirimposhi | Central | Chibombo |
| Copperbelt | Lufwanyama | Copperbelt | Masaiti | Copperbelt | Mpongwe |
| Eastern | Lundazi | Eastern | Nyimba | Eastern | Chadiza |
| Luapula | Mwense | Luapula | Kawambwa | Luapula | Milenge |
| Northern | Mporokoso | Northern | Chilubi | Northern | Chinsali |
| Northern | Isoka | Northern | Nakonde | Northern | Mpulungu |
| Northwestern | Mufumbwe | Northwestern | Mwinilunga | Northwestern | Chavuma |
| Southern | Siavonga | Southern | Namwala | Southern | Mazabuka |
| Southern | Gwembe | Southers | Itezhi-tezhi | Southern | Kazungula |
| Western | Senanga | Western | Kalabo | Western | Shangombo |

The number of health facilities in these districts is as follows:

| Province | RBF Districts | HFs | C1 Districts | HFs | C2 Districts | HFs |
| --- | --- | --- | --- | --- | --- | --- |
| Central | Mumbwa | 27 | Kapiri Mposhi | 29 | Chibombo | 33 |
| Copperbelt | Lufwanyama | 17 | Masaiti | 21 | Mpongwe | 14 |
| Eastern | Lundazi | 38 | Nyimba | 17 | Chadiza | 16 |
| Luapula | Mwense | 20 | Kawambwa | 21 | Milenge | 8 |
| Northern | Isoka | 8 | Nakonde | 10 | Mpulungu | 11 |
|  | Mporokoso | 11 | Chilubi | 11 | Chinsali | 16 |
| North-Western | Mufumbwe | 11 | Mwinilunga | 19 | Chavuma | 7 |
| Southern | Siavonga | 13 | Namwala | 12 | Mazabuka | 37 |
|  | Gwembe | 10 | Itezhi-tezhi | 14 | Kazungula | 20 |
| Western | Senanga | 20 | Kalabo | 19 | Shangombo | 13 |
| Total HFs |  | 175 |  | 173 |  | 175 |

And the population in these districts is as follows:

| Province | RBF Districts | Pop. | C1 Districts | Pop. | C2 Districts | Pop. |
| --- | --- | --- | --- | --- | --- | --- |
| Central | Mumbwa | 226,171 | Kapiri Mposhi | 253,786 | Chibombo | 303,519 |
| Copperbelt | Lufwanyama | 78,503 | Masaiti | 103,857 | Mpongwe | 93,380 |
| Eastern | Lundazi | 323,870 | Nyimba | 85,025 | Chadiza | 107,327 |
| Luapula | Mwense | 119,841 | Kawambwa | 134,414 | Milenge | 43,337 |
| Northern | Mporokoso | 98,842 | Chilubi | 81,248 | Chinsali | 146,518 |
|  | Isoka | 72,189 | Nakonde | 119,708 | Mpulungu | 98,073 |
| North-Western | Mufumbwe | 58,062 | Mwinilunga | 104,317 | Chavuma | 35,041 |
| Southern | Siavonga | 90,213 | Namwala | 102,866 | Mazabuka | 230,972 |
|  | Gwembe | 53,117 | Itezhi-tezhi | 68,599 | Kazungula | 104,731 |
| Western | Senanga | 126,506 | Kalabo | 128,904 | Shangombo | 93,303 |
| Subtotal Pop. |  | 1,247,314 |  | 1,182,724 |  | 1,256,201 |
|  | Katete | 243,849 |  |  |  |  |
| Total Pop. |  | 1,491,163 |  |  |  |  |

Note: All populations are based on the 2010 Census of Population and Housing. Isoka and Mwinilunga districts were spilt in two in 2011.

Overall, districts selected for the PRB program evaluation study were intended to represent the median population health, socio-economic condition, and health governance capacity for the collection of districts in the provinces in which they are located. If the evaluation instead focused on exceptionally high (or exceptionally low) capacity or condition districts then this will in turn overstate (or understate) the estimate of a national scale-up for the RBF, and the project team naturally wants to avoid this possibility.
