# Supplementary material for: Incentives to change: effects of performance-based financing on health workers in Zambia
Source: Hum Resour Health. 2017 Feb 28;15:20. doi: 10.1186/s12960-017-0179-2 (PMC5331731; doi:10.1186/s12960-017-0179-2)
Supplement: Additional file 3: — Determination of performance bonuses to individual staff members of health centers. (DOCX 139 kb) [file 12960_2017_179_MOESM3_ESM.docx]

**Additional file 3. Determination of performance bonuses to individual staff members of health centers**

The Zambia PBF model is a FFS model. Bonus payments are linked not just to overall health center performance, but also to individual staff performance. Two features are built into the RBF intervention. First, each health center staff signed a “motivation contract” in RBF districts. Motivation contract establishes the terms of conditional remuneration so that employees and head of health centres have a mutual understanding that bonuses are based on individual performance and official job title. The staff in the PBF health centers are thus aware that they themselves and the health center as a whole are being evaluated that is initially targeting nine key health facility indicators found in the HMIS and are deemed as critical in improving maternal and child health services. The selection of these indicators was discussed at length and agreed upon by the MOH and its partners. The incentivized indicators are:

- Curative consultations
- Institutional deliveries by skilled birth attendant
- ANC prenatal and follow up visits
- Postnatal visit
- Full immunization of children under one year old
- Pregnant women receiving 3 doses of malaria IPT
- FP users of modern methods at the end of the month
- Pregnant women counselled and tested for HIV
- HIV exposed babies administered with Niverapine and AZT

Based on these indicators, the following health centre fee schedule as outlined in as developed:

| **No** | **Indicator** | **Index** | **Fee (Kwacha** |
| --- | --- | --- | --- |
| 1 | Curative Consultation | 1 | 1,000 |
| 2 | Institutional Deliveries by Skilled Birth Attendant | 20 | 32,000 |
| 3 | ANC prenatal and follow up visits | 5 | 8,000 |
| 4 | Postnatal visit | 10 | 16,500 |
| 5 | Full immunization of children under 1 | 7 | 11,500 |
| 6 | Pregnant women receiving 3 doses of malaria IPT | 5 | 8,000 |
| 7 | FP users of modern methods at the end of the month | 2 | 3,000 |
| 8 | Pregnant women counselled and tested for HIV |  | 9,000 |
| 9 | HIV Exposed Babies administered with Niverapine and AZT |  | 10,000 |

This fee schedule consists of the core indicators, an index (weighting) attached to each indicator and the respective fee. In arriving at the index, due consideration was accorded to the significance of the indicator, i.e. the associated result in attaining the MCH national and programme goals as well as the level of complexity in conducting the requisite intervention. For example; a curative consultation has an index of 1, where as an institutional delivery is accorded 20 – indicating that a delivery is assumed to be 20 times more complex (i.e. with regard to time taken, level of skill, drugs and supplies utilised, equipment used and immediate post delivery services) than an average curative consultation conducted.

In order to avoid a situation where these incentivized indicators become the centre of attention and improve at the detriment of all other health indicators, the non-incentivized indicators were also be routinely monitored. The aim is to ensure that improvements in indicators are comprehensive and not biased towards the incentivized ones. Therefore, if non-incentivized indicators fall below 80 percent of the expected trend (based on historical data) at any time during the RBF implementation period, the health center must meet with the DMO to review the situation and define corrective measures. If the downward trend continues, the DMO reserves the right to nullify the performance contract.

Second, the formula used to calculate individual staff incentive payments is:

(Staff Index) X (Total Amount Available for Staff Performance Incentives) X

(Percentage Score from Individual Evaluation)

(Zambia Ministry of Health, 2011)^[[1]](#footnote-1)^.

As can be seen in the table below, the staff indices vary by occupational category/job title.

Health Centre Staff Categories with Basic Salaries and Indices for Performance Bonuses

| **Categories** | **Fixed Basic Monthly Salary** | **Indices for performance bonus** |
| --- | --- | --- |
| A1:Clinical Officer | $ 450 | 0.24 |
| A1: Registered Nurse | $ 450 | 0.24 |
| A2: Enrolled Nurse | $ 320 | 0.17 |
| A2: EHT | $ 315 | 0.17 |
| A4: Data Entry Clerk | $ 150 | 0.08 |
| A5:CDE | $ 120 | 0.06 |
| A6: Security Guard | $ 80 | 0.04 |
| **Total** | **$1885.00** | **1.0** |

Every quarter each staff member is evaluated by the head of the health centre. The head of the health centre uses a score card to document health center employee performance on a scale of 0 to 100 based on conscientiousness to work, team spirit, technical skill and adaptability to work, personal development will, and contribution to overall institutional performance. The DMO or the general hospital comes up with the quality score for each health center, which in turn is factored into the calculation of the total amount available for staff performance incentives. In sum, remuneration was based on a staff index, total amount available to each health center, and individual evaluation.

Incentive distribution is determined based on individual and institutional factors. We did not exclude any the occupation categories in the table above from our analysis because they all play a role in the bonuses the health center as a whole earned, even if some of them played a tangential role in delivering MCH services.

1. Zambia Ministry of Health. Operational Implementation Manual for Results Based Financing (RBF) in Pilot District in Zambia. November 2011. [↑](#footnote-ref-1)
