# Supplementary material for: Incentives to change: effects of performance-based financing on health workers in Zambia
Source: Hum Resour Health. 2017 Feb 28;15:20. doi: 10.1186/s12960-017-0179-2 (PMC5331731; doi:10.1186/s12960-017-0179-2)
Supplement: Additional file 4: — Description of motivation and job satisfaction constructs and variables [53]. (DOCX 135 kb) [file 12960_2017_179_MOESM4_ESM.docx]

**Additional file 4. Description of motivation and job satisfaction constructs and variables**

We treated each survey question for which individuals responded to on a five-point Likert scale from 1 (least motivated/satisfied) to 5 (highly motivated/satisfied) as an ordinal variable. We also consider a construct to be made up of two or more variables. We created the motivation or job satisfaction constructs in two steps: each variable in the construct was normalized by 100%, then a mean score of all the normalized variables was estimated as the final construct score [64]. This resulted in scores for eight motivation constructs: team work, autonomy, recognition, change, self-concept, work environment, leadership, and well-being. In a parallel fashion, this resulted in scores for six constructs of job satisfaction: relationship outside the health center, relationship within the health center, work conditions, recognition, opportunities, and satisfaction with compensation. The survey contains a question asking respondents to rate their overall satisfaction with their job on a scale from 1 to 5, which we consider as an overall job satisfaction variable. The lists of 39 variables making up the eight motivation constructs and of 19 variables making up the six job satisfaction constructs are as follows:

**Motivation constructs***

| Question | | Construct |
| --- | --- | --- |
| 10.01 | Staff willingly share their expertise with other members. | Team work |
| 10.02 | When disagreements occur among staff, they try to act like peacemakers to resolve the situation themselves. | Team work |
| 10.03 | Staff willingly give their time to help each other out when someone falls behind or has difficulties with work. | Team work |
| 10.04 | Staff talk to each other before taking an action that might affect them. | Team work |
| 10.05 | Staff take steps to prevent problems arising between them. | Team work |
| 10.07 | Staff spend their time chatting amongst themselves about things that are not related to work. | Team work |
| 10.08 | Staff spend time complaining about work-related issues. | Team work |
| 10.09 | My job allows me freedom in how I organize my work and the methods and approaches to use. | Autonomy |
| 10.10 | I am given enough authority by my supervisors to do my job well. | Autonomy |
| 10.11 | It is important for me that the community recognizes my work as a professional. | Recognition |
| 10.12 | It is important for me that my peers recognize my work as a professional. | Recognition |
| 10.13 | Changes in the facility are easy to adjust to. | Change |
| 10.14 | Rapid changes are difficult to cope with. | Change |
| 10.15 | Changes bring opportunities to make improvements in the facility. | Change |
| 10.16 | My job makes me feel good about myself. | Self-concept |
| 10.17 | I am proud of the work I'm doing in this facility. | Self-concept |
| 10.22 | I complete my tasks efficiently and effectively. | Self-concept |
| 10.23 | I am a hard worker. | Self-concept |
| 10.24 | I am punctual about coming to work. | Self-concept |
| 10.25 | These days, I feel motivated to work as hard as I can. | Self-concept |
| 10.18 | I am proud to be working for this health facility. | Work environment |
| 10.19 | I am glad that I am working for this facility rather than in other facilities in the country. | Work environment |
| 10.20 | I would prefer to work somewhere else than in this facility. | Work environment |
| 10.21 | This health facility inspires me to do my very best on the job. | Work environment |
| 10.26 | My facility is a very personal place. It is like an extended family and people share a lot with each other. | Work environment |
| 10.27 | My facility is very dynamic and an innovative place. People are willing to take risks to do a job well-done. | Work environment |
| 10.28 | My facility is very formal and structured. Policies and procedures are important for doing our work. | Work environment |
| 10.29 | In my facility, we focus on achieving daily goals getting our work done. Relationships between staff are less important. | Work environment |
| 10.35 | Innovation and being first to try something new are important in my facility. | Work environment |
| 10.36 | Following procedures and rules is very important in my facility. | Work environment |
| 10.37 | Achieving results and high performance is very important in my facility. | Work environment |
| 10.30 | The head of my facility is a mentor and a role model. | Leadership |
| 10.31 | The head of my facility is willing to innovate and take risks in order to improve things. | Leadership |
| 10.33 | The head of my facility motivates staff to achieve goals. | Leadership |
| 10.38 | In the past two weeks, I have felt cheerful and in good spirits….. | Well-being |
| 10.39 | In the past 2 weeks, I have felt calm and relaxed… | Well-being |
| 10.40 | In the past 2 weeks, I have felt active and vigorous… | Well-being |
| 10.41 | In the past 2 weeks, I woke up feeling fresh and rested… | Well-being |
| 10.42 | In the past two weeks, my daily life has been filled with things that interest me…. | Well-being |

*Note: Motivation-related questions come from Section 8 ‘WHO Well-Being Index’ and Section 10 ‘Personal Drive’ of the Health Worker Individual Questionnaire. Results for these eight constructs are reported in Table 4, and for the 39 questions in Appendix 5. The first column of the table denotes the question numbers in the Zambian PBF survey questionnaire for ease of reference.

**Job satisfaction constructs***

| Question | | Construct |
| --- | --- | --- |
| 9.02 | Working relationships with District/ Ministry of Health staff | Relationship outside facility |
| 9.12 | The relationships between the health facility and local traditional leaders | Relationship outside facility |
| 9.01 | Working relationships with other facility staff | Relationship within facility |
| 9.03 | Working relationships with Management staff within the health facility | Relationship within facility |
| 9.05 | Quantity of medicine available in the health facility | Work conditions |
| 9.07 | Quantity of equipment in the health facility | Work conditions |
| 9.08 | Quality and physical condition of equipment in the health facility | Work conditions |
| 9.09 | Availability of other supplies in the health facility (compresses, etc.; office supplies) | Work conditions |
| 9.10 | The physical condition of the health facility building | Work conditions |
| 9.11 | Your ability to provide high quality of care given the current working conditions in the facility | Work conditions |
| 9.13 | Your level of respect in the community | Recognition |
| 9.16 | Your immediate supervisor's recognition of your good work | Recognition |
| 9.14 | Your opportunities to upgrade your skills and knowledge through training | Opportunities |
| 9.15 | Your opportunity to discuss work issues with your immediate supervisor | Opportunities |
| 9.18 | The opportunities to use your skills in your job. | Opportunities |
| 9.21 | Your opportunities for promotion | Opportunities |
| 9.17 | Your opportunity to be rewarded for hard work, financially or otherwise. | Compensation |
| 9.19 | Your salary | Compensation |
| 9.20 | Your benefits (such as housing, travel allowance, bonus including performance bonus, etc) | Compensation |
| 9.25 | Overall, how satisfied are you with your job? | Overall satisfaction |

*Note: Satisfaction-related questions come from Section 9 ‘Health Worker Satisfaction’ of the Health Worker Individual Questionnaire. Results for the six constructs are reported in Table 5, and for the 20 questions in Appendix 6. The first column of the table denotes the question numbers in the Zambian PBF survey questionnaire for ease of reference.
