# Supplementary material for: Incentives to change: effects of performance-based financing on health workers in Zambia
Source: Hum Resour Health. 2017 Feb 28;15:20. doi: 10.1186/s12960-017-0179-2 (PMC5331731; doi:10.1186/s12960-017-0179-2)
Supplement: Additional file 5: — Mean statistics of health worker characteristics at baseline and endline, two-group comparisons. (DOCX 89 kb) [file 12960_2017_179_MOESM5_ESM.docx]

**Additional file 5. Mean statistics of health worker characteristics at baseline and endline, two-group comparisons**

**Table of comparison of treatment group and control 1 group characteristics**

|  | **Baseline** | | | **Endline** | | |
| --- | --- | --- | --- | --- | --- | --- |
| **Variable** | **Control 1 mean** | **PBF mean** | **p-value** | **Control 1 mean** | **PBF mean** | **p value** |
| Female | 0.38 | 0.42 | 0.53 | 0.36 | 0.41 | 0.46 |
| Education-primary | 0.08 | 0.06 | 0.57 | 0.01 | 0.04 | 0.17 |
| Education-secondary | 0.40 | 0.40 | 0.90 | 0.49 | 0.35 | **0.03** |
| Education-college | 0.49 | 0.52 | 0.64 | 0.49 | 0.60 | *0.08* |
| Clinical officer | 0.02 | 0.03 | 0.63 | 0.04 | 0.06 | 0.53 |
| Nurse | 0.26 | 0.25 | 0.90 | 0.35 | 0.33 | 0.72 |
| Midwife | 0.13 | 0.11 | 0.69 | 0.09 | 0.12 | 0.45 |
| Environmental Health Technicians | 0.09 | 0.15 | 0.25 | 0.08 | 0.13 | 0.23 |
| Classified Daily Employees | 0.41 | 0.33 | 0.22 | 0.38 | 0.31 | 0.28 |
| Other staff | 0.59 | 0.67 | 0.22 | 0.62 | 0.69 | 0.28 |
| Age | 38.01 | 37.43 | 0.68 | 38.51 | 35.82 | **0.04** |
| Work-absence | 1.44 | 1.20 | 0.69 | 1.10 | 1.12 | 0.96 |
| Work-days | 6.26 | 5.82 | *0.04* | 6.24 | 6.00 | 0.23 |
| Work-hrs | 55.90 | 51.45 | 0.34 | 50.33 | 52.07 | 0.59 |
| Supervision | 4.32 | 4.52 | 0.80 | 4.58 | 5.62 | 0.16 |
| Work experience-total | 11.04 | 10.06 | 0.48 | 9.03 | 8.03 | 0.39 |
| Work experience-current facility | 5.40 | 4.55 | 0.27 | 4.67 | 4.27 | 0.55 |

Note: Statistical significance is denoted by: bold italic (p<0.01); bold (p<0.05); italic (p<0.1).

**Table of comparison of treatment group and control 2 group characteristics**

|  | **Baseline** | | | **Endline** | | |
| --- | --- | --- | --- | --- | --- | --- |
| **Variable** | **Control 2 mean** | **PBF mean** | **p-value** | **Control 2 mean** | **PBF mean** | **p-value** |
| Female | 0.42 | 0.42 | 0.97 | 0.49 | 0.41 | 0.21 |
| Education-primary | 0.05 | 0.06 | 0.82 | 0.05 | 0.04 | 0.75 |
| Education-secondary | 0.30 | 0.40 | 0.12 | 0.27 | 0.35 | 0.20 |
| Education-college | 0.63 | 0.52 | *0.10* | 0.68 | 0.60 | 0.23 |
| Clinical officer | 0.04 | 0.03 | 0.73 | 0.03 | 0.06 | 0.43 |
| Nurse | 0.25 | 0.25 | 0.98 | 0.45 | 0.33 | *0.06* |
| Midwife | 0.14 | 0.11 | 0.48 | 0.15 | 0.12 | 0.58 |
| Environmental Health Technicians | 0.16 | 0.15 | 0.71 | 0.10 | 0.13 | 0.55 |
| Classified Daily Employees | 0.32 | 0.33 | 0.77 | 0.22 | 0.31 | 0.11 |
| Other staff | 0.69 | 0.67 | 0.77 | 0.78 | 0.69 | 0.11 |
| Age | 36.21 | 37.43 | 0.37 | 35.49 | 35.82 | 0.80 |
| Work-absent | 1.59 | 1.20 | 0.46 | 1.74 | 1.12 | 0.22 |
| Work-days | 6.13 | 5.82 | 0.18 | 6.27 | 6.00 | 0.13 |
| Work-hrs | 54.55 | 51.45 | 0.52 | 49.61 | 52.07 | 0.38 |
| Supervision | 6.65 | 4.52 | 0.26 | 4.54 | 5.62 | 0.16 |
| Work experience-total | 9.76 | 10.06 | 0.82 | 7.95 | 8.03 | 0.94 |
| Work experience-current facility | 4.39 | 4.55 | 0.81 | 5.09 | 4.27 | 0.21 |

Note: Statistical significance is denoted by: bold italic (p<0.01); bold (p<0.05); italic (p<0.1).

**Table of comparison of control I group and control II group characteristics**

|  | **Baseline** | | | **Endline** | | |
| --- | --- | --- | --- | --- | --- | --- |
| **Variable** | **C2 mean** | **C1 mean** | **p value** | **C2 mean** | **C1 mean** | **p value** |
| Female | 0.42 | 0.38 | 0.55 | 0.49 | 0.36 | *0.08* |
| Education-primary | 0.05 | 0.08 | 0.48 | 0.05 | 0.01 | 0.12 |
| Education-secondary | 0.30 | 0.40 | 0.21 | 0.27 | 0.49 | ***0.00*** |
| Education-college | 0.63 | 0.49 | *0.06* | 0.68 | 0.49 | ***0.01*** |
| Clinical officer | 0.04 | 0.02 | 0.46 | 0.03 | 0.04 | 0.90 |
| Nurse | 0.25 | 0.26 | 0.93 | 0.45 | 0.35 | 0.19 |
| Midwife | 0.14 | 0.13 | 0.80 | 0.15 | 0.09 | 0.24 |
| Environmental Health Technicians | 0.16 | 0.09 | 0.17 | 0.10 | 0.08 | 0.55 |
| Classified Daily Employees | 0.32 | 0.41 | 0.17 | 0.22 | 0.38 | **0.02** |
| Other staff | 0.69 | 0.59 | 0.17 | 0.78 | 0.62 | **0.02** |
| Age | 36.21 | 38.01 | 0.22 | 35.49 | 38.51 | *0.06* |
| Work-absent | 1.59 | 1.44 | 0.84 | 1.74 | 1.10 | 0.32 |
| Work-days | 6.13 | 6.26 | 0.50 | 6.27 | 6.24 | 0.87 |
| Work-hrs | 54.55 | 55.90 | 0.79 | 49.61 | 50.33 | 0.83 |
| Supervision | 6.65 | 4.32 | 0.31 | 4.54 | 4.58 | 0.96 |
| Work experience-total | 9.76 | 11.04 | 0.36 | 7.95 | 9.03 | 0.45 |
| Work experience-current facility) | 4.39 | 5.40 | 0.22 | 5.09 | 4.67 | 0.61 |

Note: Statistical significance is denoted by: bold italic (p<0.01); bold (p<0.05); italic (p<0.1).
