# Supplementary material for: Incentives to change: effects of performance-based financing on health workers in Zambia
Source: Hum Resour Health. 2017 Feb 28;15:20. doi: 10.1186/s12960-017-0179-2 (PMC5331731; doi:10.1186/s12960-017-0179-2)
Supplement: Additional file 6: — Regression results for motivation variables. (DOCX 82 kb) [file 12960_2017_179_MOESM6_ESM.docx]

**Additional file 6. Regression results for motivation variables**

| Construct | Question | Intervention v. control 1 N=448 | Intervention v. control 2 N=462 | Control 1 v. control 2 N=345 |
| --- | --- | --- | --- | --- |
|  |  | β (standard error) | β (standard error) | β (standard error.) |
| Teamwork | 10.01 | 0.53 (3.18) | 0.76 (1.65) | 0.91 (3.13) |
|  | 10.02 | 6.31 (4.50) | 2.72 (1.91) | -0.44 (5.33) |
|  | 10.03 | 3.72 (3.95) | 3.77** (1.48) | 3.53 (4.31) |
|  | 10.04 | -0.31 (4.23) | 0.67 (2.80) | 2.22 (5.31) |
|  | 10.05 | 2.12 (4.70) | 1.45 (2.46) | 0.96 (3.69) |
|  | 10.07 | -1.98 (5.99) | -0.54 (3.57) | 0.76 (8.48) |
|  | 10.08 | -7.99 (8.31) | -2.40 (3.80) | 3.63 (9.21) |
| Autonomy | 10.09 | 3.06 (5.84) | 1.05 (2.54) | -1.55 (6.34) |
|  | 10.10 | -1.23 (3.83) | 1.63 (1.44) | 4.14 (3.91) |
| Recognition | 10.11 | -0.68 (3.11) | -0.69 (1.20) | -0.34 (2.97) |
|  | 10.12 | -0.21 (3.81) | -1.06 (1.65) | -1.44 (2.95) |
| Change | 10.13 | 0.78 (4.43) | -0.26 (2.11) | -1.45 (3.71) |
|  | 10.14 | -4.64 (6.32) | 3.01 (3.72) | 9.79 (7.34) |
|  | 10.15 | -2.43 (3.73) | 0.33 (2.05) | 3.15 (4.02) |
| Self concept | 10.16 | -1.07 (3.11) | 0.84 (1.80) | 2.61 (2.74) |
|  | 10.17 | -2.26 (2.59) | 0.57 (1.25) | 3.24 (2.86) |
|  | 10.22 | -3.42 (4.27) | 0.69 (1.40) | 4.56 (4.16) |
|  | 10.23 | -4.20 (2.43) | -0.23 (0.63) | 3.57 (2.43) |
|  | 10.24 | 0.34 (2.62) | -1.21 (1.72) | -2.79 (3.49) |
|  | 10.25 | 7.18 (4.17) | 4.09 (2.45) | 1.46 (5.11) |
| Work Environment | 10.18 | -3.14 (4.71) | 1.66 (1.72) | 6.51 (4.76) |
|  | 10.19 | -8.73 (5.79) | -2.25 (2.70) | 4.87 (6.59) |
|  | 10.20 | -12.27 (6.86) | -3.45 (2.69) | 5.27 (6.80) |
|  | 10.21 | -0.25 (6.92) | 3.10 (3.25) | 6.43 (7.16) |
|  | 10.26 | 1.16 (6.77) | 1.75 (3.08) | 2.82 (6.18) |
|  | 10.27 | 2.92 (6.19) | 5.06* (2.65) | 7.22 (7.12) |
|  | 10.28 | 1.91 (3.59) | 2.24 (1.76) | 2.56 (3.97) |
|  | 10.29 | -2.73 (5.93) | 0.04 (2.71) | 2.54 (6.22) |
|  | 10.35 | -1.09 (4.48) | 1.11 (1.85) | 3.33 (3.52) |
|  | 10.36 | -0.71 (2.31) | 1.95* (1.02) | 4.26* (2.09) |
|  | 10.37 | 1.24 (2.41) | 1.32 (1.05) | 1.06 (2.31) |
| Leadership | 10.30 | -4.27 (4.97) | 0.17 (2.59) | 4.79 (6.05) |
|  | 10.31 | -1.74 (6.05) | 2.46 (2.70) | 6.46 (5.94) |
|  | 10.33 | -3.21 (5.24) | 1.02 (3.10) | 5.40 (5.11) |
| Well-being | 10.38 | -1.73 (5.08) | 1.89 (1.67) | 5.66 (4.38) |
|  | 10.39 | 9.48 (5.46) | 5.69** (2.38) | 1.86 (4.46) |
|  | 10.40 | -3.60 (4.23) | -1.04 (1.61) | 1.82 (4.97) |
|  | 10.41 | 2.23 (4.49) | 1.35 (2.42) | 0.34 (5.47) |
|  | 10.42 | -0.88 (6.15) | 4.20* (2.26) | 9.99 (5.96) |

*Note: The question numbers in Appendix 4 are the same as those that appear in Appendix 3. Coefficients, standard errors, and p-values are for the interaction between the random assignment (intervention, control 1, control 2) and study period (baseline, endline). They are obtained from pair-wise regressions—facility fixed effect models controlling for workers’ characteristics. Robust standard errors are clustered at facility level.

* p<0.1; ** p<0.05
