# Supplementary material for: Incentives to change: effects of performance-based financing on health workers in Zambia
Source: Hum Resour Health. 2017 Feb 28;15:20. doi: 10.1186/s12960-017-0179-2 (PMC5331731; doi:10.1186/s12960-017-0179-2)
Supplement: Additional file 7: — Regression results for job satisfaction variables. (DOCX 83 kb) [file 12960_2017_179_MOESM7_ESM.docx]

**Additional file 7. Regression results for job satisfaction variables**

| Construct | Question | Intervention v. control 1 (N=448) | Intervention v. control 2 (N=462) | Control 1 v. control 2 (N=345) |
| --- | --- | --- | --- | --- |
|  |  | β (standard error) | β (standard error) | β (standard error) |
| Relationship outside facility | 9.02 | 1.66 (5.41) | 1.96 (2.08) | 2.67 (5.39) |
|  | 9.12 | 1.63 (4.65) | -1.10 (1.95) | -3.85 (5.31) |
| Relationship within facility | 9.01 | -6.76** (3.23) | -0.47 (1.25) | 5.51 (3.37) |
|  | 9.03 | -1.70 (3.35) | 1.50 (1.61) | 4.71 (3.61) |
| Work conditions | 9.05 | 7.42 (7.43) | 2.44 (3.36) | -2.63 (8.64) |
|  | 9.07 | 2.18 (7.26) | 4.42 (3.25) | 6.46 (7.02) |
|  | 9.08 | 8.91 (7.02) | 5.04* (2.84) | 1.37 (6.99) |
|  | 9.09 | 12.97* (7.43) | 7.73** (2.99) | 2.04 (8.29) |
|  | 9.10 | 7.06 (8.40) | 4.98* (2.77) | 2.48 (8.91) |
|  | 9.11 | -0.18 (7.09) | 1.59 (3.55) | 3.47 (6.96) |
| Recognition | 9.13 | 5.78 (4.25) | 0.09 (2.09) | -5.73* (3.24) |
|  | 9.16 | -2.90 (3.75) | 0.08 (2.37) | 2.86 (4.11) |
| Opportunities | 9.14 | 12.00** (4.98) | 7.02* (3.66) | 1.67 (7.48) |
|  | 9.15 | 1.85 (5.37) | 1.32 (2.48) | 0.03 (5.82) |
|  | 9.18 | -2.23 (4.56) | 3.36 (2.70) | 8.91 (6.19) |
|  | 9.21 | 6.73 (6.94) | 2.34 (2.95) | -2.09 (7.55) |
| Compensation | 9.17 | 6.88 (5.46) | 8.88*** (2.89) | 10.85* (6.13) |
|  | 9.19 | 6.59 (5.18) | 1.33 (2.30) | -3.72 (6.23) |
|  | 9.20 | 12.82* (6.40) | 1.69 (3.07) | -9.51 (7.80) |
| Overall satisfaction | 9.25 | -0.48 (3.96) | 4.75** (2.14) | 10.31** (3.94) |

Note: The question numbers in Appendix 5 are the same as those that appear in Appendix 3. Coefficients, standard errors, and p-values are for the interaction between the random assignment (intervention, control 1, control 2) and study period (baseline, endline). They are obtained from pair-wise regressions—facility fixed effect models controlling for workers’ characteristics. Robust standard errors are clustered at facility level.

* p<0.1; ** p<0.05
